# Dietary Intake and Diet Quality of Female and Male NCAA Division I Cross Country Runners from a Single University

**DOI:** 10.1016/j.cdnut.2024.104475

**Published:** 2024-10-15

**Authors:** David E Barney, Susan N Cheung, Aaron R Harris, Claire E Berryman, Stephen R Hennigar

**Affiliations:** 1 Department of Nutrition and Integrative Physiology, Florida State University, Tallahassee, FL, United States; 2 Pennington Biomedical Research Center, Baton Rouge, LA, United States; 3 Military Nutrition Division, United States Army Research Institute of Environmental Medicine, Natick, MA, United States; 4 Oak Ridge Institute for Science and Education, Belcamp, MD, United States

**Keywords:** cross country, diet, HEI-2020, runners, student-athletes

## Abstract

**Background:**

Collegiate student-athletes have unique nutritional requirements to support their athletic performance and health. Few studies have comprehensively characterized the diets of National Collegiate Athletic Association (NCAA) Division I student-athletes.

**Objectives:**

To characterize dietary intake and diet quality during a competitive season in female and male NCAA Division I cross country student-athletes from a single university.

**Methods:**

Females and males (*n* = 14/sex) from the Florida State University cross country teams completed 9-d of food records across their competitive season. Nutrient intakes were compared to the Dietary Reference Intakes for the United States population [e.g., Recommended Daily Allowances (RDAs)] and athlete-specific guidelines. Diet quality was assessed according to the Dietary Guidelines for Americans (DGAs) using the 2020 Healthy Eating Index (HEI-2020). Total daily energy expenditure was estimated from training records.

**Results:**

Carbohydrate intakes were below athlete guidelines in 43% of females (*mean* ± *SD*, 5.67 ± 1.16 g·kg^–1^·d^–1^) and 29% of males (4.95 ± 1.05, *P sex* = 0.096). All participants met or exceeded athlete recommendations for protein (2.09 ± 0.425 g·kg^–1^·d^–1^, 1.92 ± 0.519, *P sex* = 0.36) and fat (32.8 ± 5.1% kcal, 34.4 ± 3.4%, *P sex* = 0.36). No participants met the RDA for vitamin D (5.14 ± 1.78 μg/d, 4.91 ± 3.24, *P sex* = 0.83). Only 79% of females and 36% of males met the RDA for calcium (1220 ± 307 mg/d, 1010 ± 296, *P sex* = 0.83). Most females (*n* = 13) and males (*n* = 11) consumed iron supplements where total intakes exceeded the tolerable upper intake level (110 ± 60.1 mg/d, 66.8 ± 36.3, *P sex* = 0.029). HEI-2020 indicated poor adherence to the DGAs, with better diet quality in females (65.3 ± 13.7) than males (50.6 ± 10.1, *P sex* = 0.0034). Participants failed to meet guidelines for all HEI-2020 food group components except total protein foods. Total daily energy expenditure was greater in males and declined across the competitive season (*P sex* < 0.0001, *P time* < 0.0001, *P sex∗time* = 0.25).

**Conclusions:**

NCAA Division I cross country student-athletes consumed inadequate carbohydrates, calcium, and vitamin D but met or exceeded intake guidelines for protein, fat, and iron. Diet quality was poor; HEI-2020 component scores may indicate food groups to target to improve diet quality and intake of nutrients important to runners.

This trial was registered at clinicaltrials.gov as NCT04079322.

## Introduction

Nutrition guidance for the United States population is provided by the Dietary Reference Intakes (DRIs) and the Dietary Guidelines for Americans (DGAs). Published since 1998 by the Food and Nutrition Board of the National Academies, the DRIs provide age- and sex-specific nutrient intakes aimed to ensure most healthy Americans consume nutritionally adequate diets while minimizing risk for adverse health outcomes and chronic disease [1]. The DGAs are released every 5 y by the US Department of Agriculture (USDA) and Health and Human Services and provide a general framework for Americans to consume healthy dietary patterns across all ages, cultural and financial backgrounds, and personal preferences [2]. While the DRIs set recommended intakes of specific nutrients, the DGAs give quantitative guidance on foods to consume for a healthy dietary pattern. According to the DGAs, a healthy dietary pattern meets food group needs with nutrient-dense foods and beverages within calorie limits and limits foods and beverages high in saturated fat, added sugars, and sodium [2]. Together, the DRIs and DGAs serve as the foundational evidence base for clinicians, scientists, and policymakers in nutrition.

Athletes and other highly physically active populations have unique nutritional requirements that are not fully addressed by DRIs and DGAs. The American College of Sports Medicine (ACSM), the Academy of Nutrition and Dietetics (AND), and the Dietitians of Canada (DC) jointly provide nutrition guidelines to support performance and health in competitive athletes and active adults [3]. Endurance athletes, in particular, have some of the highest dietary intake requirements to fuel their high training volumes [4], increasing the risk for energy and nutrient deficiencies. To provide adequate nourishment for training, competition, and recovery, ACSM/AND/DC guidelines recommend athletes with high physical activity levels to consume carbohydrate and protein intakes above the DRIs [3]. Athletes with high physical activity levels are also recommended to meet or exceed the DRIs for select micronutrients, including vitamin D, calcium, and iron, particularly in athletes at risk for energy deficiency (i.e., negative energy balance or low energy availability) [3]. Despite recommendations, recent studies indicate that collegiate student-athletes have poor knowledge of sports nutrition [[5], [6], [7], [8]], often do not meet nutrient intake guidelines for athletes [[7], [8], [9], [10]], and have poor diet quality according to the DGAs [[10], [11], [12]]. In a 2022 legislative initiative to benefit student-athlete health and wellness, the National Collegiate Athletic Association (NCAA) lifted restrictions on when and how often Division I athletic departments can provide meals, snacks, and nutritional supplements to student-athletes during a competitive season [13]. This policy shift greatly increases possible intervention points to improve the diets of NCAA Division I student-athletes. Nutrition education programs, in particular, appear effective at improving dietary intake in athletes [14], and the NCAA recently identified nutrition as the top area in health and wellness, where student-athletes request more support from coaches and administrators [15]. However, evidence on how to intervene is limited, as only a small number of studies have assessed dietary intake in Division I student-athletes, especially cross country runners (reviewed in Supplemental Table 1) [[7], [8], [9], [10], [11], [12],[16], [17], [18]].

The purpose of the current study was to characterize dietary intake during a competitive season in female and male NCAA Division I cross country student-athletes. We hypothesized that macronutrient intakes would meet age-specific DRIs; however, carbohydrate and protein intakes would not meet guidelines for athletes, and diet quality assessed by the 2020 Healthy Eating Index (HEI-2020) would indicate poor adherence to the DGAs. Secondarily, we aimed to estimate energy expenditure across a competitive cross country season. We hypothesized that the estimated total daily energy expenditure (TDEE) would be greater than dietary energy intake, and the majority of participants would be estimated to have a negative energy balance (i.e., energy deficiency).

## Methods

### Participants and study design

**FIGURE 1:**
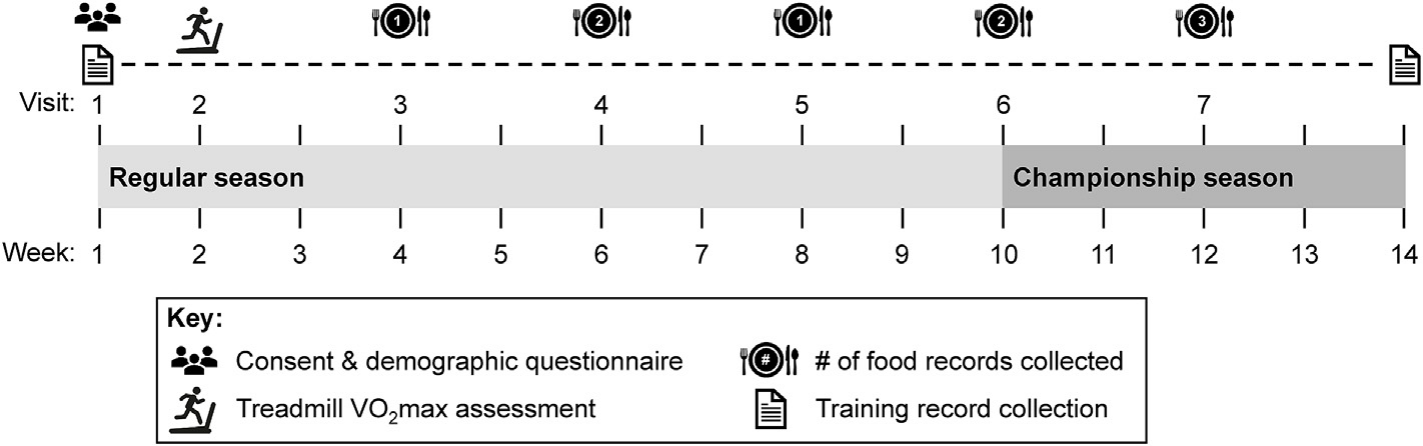
Protocol overview of dietary intake and estimated energy expenditure in NCAA Division I cross country student-athletes during a competitive season. Participants completed food records for the days preceding and days of visits 3–7, with superscripts indicating the number of days analyzed for each visit (9 d total, regular season: 4 d, championship season: 5 d). NCAA, National Collegiate Athletic Association; VO_2_max, volume of maximal oxygen uptake.

The current analysis was a secondary objective of a study primarily aimed at determining the impact of a prolonged bout of running on circulating hepcidin and dietary iron absorption [19]. The research was approved by the Florida State University institutional review board and registered at clinicaltrials.gov (NCT04079322). Participants were recruited from the Florida State University Men’s and Women’s Cross Country teams upon reporting to campus for the 2019 competitive season. Participants were eligible to participate if they had no recent history of musculoskeletal injury and were willing to refrain from dietary supplements and anti-inflammatory medications on visits pertaining to the primary aims. Immediately following the provision of written and informed consent (visit 1, Figure 1), participants completed a demographic questionnaire to assess general health, age, race/ethnicity, dietary restrictions, food allergies, and menstrual cycle regularity in females. Contraceptive use and reproductive hormone status were not assessed in females. As such, females reporting ≤35 d between periods were considered naturally menstruating [20]. Females reporting >35 d between periods were considered possibly oligomenorrheic, and females reporting no periods in the previous 3 mo were considered possibly amenorrheic. Baseline height, bodyweight, and fitness [volume of maximal oxygen uptake (VO_2_max)] were assessed 3–4 d later (visit 2), as previously described [19]. For data collection related to the primary study, participants returned to the laboratory for 5 additional visits (visits 3–7), during which food records were collected for this secondary objective. Each visit was separated by 1–3 wk across the entirety of the competitive season (Figure 1): 3 visits during the regular season and 2 visits during the championship season, defined as the week of the conference championship through the NCAA championship. From consent to the conclusion of the championship season, training records were collected to estimate energy expenditure from exercise (EEE). Fasted morning bodyweight was measured again during the championship season (visit 7). World Athletics scores were determined *post hoc* using each athlete’s best cross country and track times before or within the academic year of the study (August 2019 to July 2020). Scores were determined from the 2017 World Athletics scoring tables corresponding to the period of data collection [21].

### Dietary intake across the competitive season

Food records were given to participants ahead of time, and participants were asked to record foods as they were consumed for the 1–2 d preceding and the day of study visits. Instructions on food record completion were provided by a registered dietitian. Participants were instructed to include details such as brand names, place of purchase, cooking method, use of cooking oils and seasonings, and time of consumption. The blank food record (Supplemental Materials) included these prompts with a visual guide for serving sizes adapted from the 2014 Block Food Frequency Questionnaire (NutritionQuest). At study visits, research staff reviewed food records with participants to ensure completeness. For this secondary study, days of experimental visits during visits 3–6 were excluded to minimize a possible confounding effect of the primary intervention on dietary intake and because participants were asked to refrain from dietary supplement intake on days of experimental visits. Dietary intake data from excluded days were also previously reported [19]. In the current analysis, 9 d of food records were analyzed for each participant (regular season: 4 d, championship season: 5 d, Figure 1), except 1 female did not record for visit 4 (1 d missing) and 2 males did not record for visit 7 (3 d missing each).

Nutrient data from food records were generated in Food Processor version 11.9 (ESHA Research) and matched by nutrient composition to food and beverage items sourced from the USDA’s Food and Nutrient Database for Dietary Studies (FNDDS) and National Nutrient Database for Standard Reference, Legacy (2018, SR-Legacy) [22,23]. If exact matches were not found, similar foods or ingredients were selected from these databases based on macronutrient composition. Nutrition labels, restaurant menus, university dining hall menus, and athletic department snack station inventories were referenced to match nutrient compositions in Food Processor. As dietary supplements are not included in FNDDS and SR-Legacy, nutrients from dietary supplements were added to analyzed diet records after export from the Food Processor. Water intake was not included in the analysis. For each participant, mean nutrient intakes were calculated across all food records to determine adherence to age- and sex-specific DRIs [e.g., ≥Recommended Daily Allowances (RDAs), ≥Adequate Intake Levels (AIs), within Acceptable Macronutrient Distribution Ranges (AMDRs), >Tolerable Upper Intake Levels (ULs)] and athlete-specific recommendations from ACSM/AND/DC [1,3]. Mean intakes for all females and males were also compared against age- and sex-specific DRIs using the mean age of females and males, respectively.

### HEI-2020

HEI-2020 scores were calculated to assess diet quality according to the DGAs [2]. The HEI is updated every 5 y alongside the DGAs. This most recent iteration was published alongside the 2020–2025 DGAs [24] and maintains the same scoring criteria from HEI-2015 [25,26]. HEI-2020 provides a 100-point score of overall diet quality by scoring 13 individual dietary components, 9 of which are evaluated on adequacy in the diet (total vegetables, greens and beans, total fruits, whole fruits, whole grains, dairy, total protein foods, seafood and plant proteins, and fatty acids) and 4 of which are evaluated on moderation in the diet (sodium, refined grains, saturated fats, and added sugars). HEI-2020 component scores were calculated using nutrient data from the FNDDS and SR-Legacy databases and food group data generated by converting FNDDS and SR-Legacy items to the 37 USDA food pattern components (e.g., cup equivalents of fruit, ounce equivalents of protein foods, gram equivalents of solid fats and oils) in the Food Patterns Equivalents Database (FPED) [27]. HEI-2020 component and total scores were calculated in SAS version 9.4 using the National Cancer Institute simple scoring method, which sums components and energy across all food records per individual before applying the scoring algorithm [28]. DGA adherence is indicated by a total score of 100; component scores of 5 for total fruits, whole fruits, total vegetables, greens and beans, total protein foods, and seafood and plant protein; and component scores of 10 for whole grains, dairy, fatty acids, refined grains, sodium, added sugars, and saturated fats [[24], [25], [26]].

### Training data, estimated energy expenditure, and energy balance

Training records were collected from consent until the end of the championship season. Participants’ personal training journals were turned in to the coaching staff weekly for coaches to monitor athlete training, and journals were photocopied with consent for use by the research staff. Training journals were cross-referenced for completeness with coaches’ training plans and records of completed training sessions. Training time and intensity from each session and bodyweight were used to calculate daily EEE using metabolic equivalents from the 2011 Compendium of Physical Activities [29]. At the end of the season, an online questionnaire (Qualtrics) was administered to determine typical training habits across the competitive season, including time and intensities for running (pace), resistance training, and cross-training (cycling, swimming, etc, Supplemental Materials). Intensities were based on codes from the Compendium of Physical Activities. Thus, if training time or intensity were not provided in an athlete or coach’s record, answers from the Qualtrics survey were used to calculate EEE. TDEE was calculated for each day by adding EEE to resting energy expenditure calculated from the Mifflin-St Jeor equation [30]. Energy balance was calculated as energy intake minus TDEE. Changes in energy balance across the season were crudely estimated by grouping diet recalls into the regular season (weeks 1–10) and the championship season (weeks 10–14, Figure 1). In the online questionnaire, participants were also asked to list their nonseason-ending injuries and illnesses and how their training was impacted.

### Statistics

Statistics were performed in GraphPad Prism version 9.5. Normality was assessed with the Shapiro-Wilk test. If not normally distributed (*P* < 0.05), data were log-transformed. If log transformation did not create a normal distribution, data were analyzed by nonparametric tests. Sex differences in baseline characteristics, nutrient intake, and diet quality were assessed by unpaired t-tests and Mann-Whitney tests. Two-way repeated measures analysis of variances (ANOVAs) with main effects of sex, time, and sex∗time interactions assessed bodyweight, BMI (in kg/m^2^), TDEE, and weekly mileage across the competitive season. If an interaction effect was observed, Bonferroni’s test for multiple comparisons assessed post hoc differences. If no interaction effect was observed, repeated measures 1-way ANOVAs with Bonferroni’s test assessed differences over time within a sex. Statistical significance was set to *P* < 0.05. Data are means ± SDs.

## Results

### Participant demographics and anthropometrics

Thirty-one participants consented (15 females, 16 males), and 28 participants completed the study and were included in the final analysis (14 females, 14 males). Reasons for exclusions included departure from the team (1 female) and season-ending injuries (2 males). Participant characteristics at baseline are provided in Table 1. Participants were ∼20 y old and mostly identified as non-Hispanic White race/ethnicity. At baseline, 64% of females but no males were classified as underweight (BMI <18.5), and nearly half (43%) of females were considered possibly oligo- or amenorrheic, though it is again important to note that contraceptive use was not assessed. Females reported no dietary restrictions, whereas 2 males reported lactose sensitivity, and another male reported allergies to eggs and nuts. VO_2_max was greater in males than females (*P* = 0.0035), but World Athletics scores did not differ (*P* = 0.109). Both indicate an elite training status (tier 2 athletes per [31]).

**TABLE 1:** Characteristics of NCAA Division I cross country student-athletes at the start of a competitive season.

Characteristics	Combined (*n* = 28)	Females (*n* = 14)	Males (*n* = 14)
Demographics			
Age, y	19.7 ± 1.2	19.6 ± 1.3	19.8 ± 1.3
Academic year, *n* (%):			
Freshman	6 (21)	3 (21)	3 (21)
Sophomore	7 (25)	3 (21)	4 (29)
Junior	10 (36)	7 (50)	3 (21)
Senior	3 (11)	1 (7)	2 (14)
Graduate	2 (7)	0 (0)	2 (14)
Race/ethnicity, *n* (%)^1^:			
White	26 (93)	14 (100)	12 (86)
Black/African American	2 (7)	0 (0)	2 (14)
Hispanic or Latinx	2 (7)	0 (0)	2 (14)
Anthropometrics and menstrual status			
Height, cm	172 ± 7.6	167 ± 4.9	177 ± 6.9∗
Weight, kg	57.1 ± 8.6	50.6 ± 5.8	63.6 ± 5.4∗
BMI, kg/m^2^	19.2 ± 2.0	18.1 ± 1.6	20.4 ± 1.6∗
Menstrual regularity, *n* (%):			
Naturally menstruating	--	8 (57)	--
Possibly oligomenorrheic	--	3 (21)	--
Possibly amenorrheic	--	3 (21)	--
Training and performance status			
VO_2_max, mL‧kg^–1^‧min^–2^	66.1 ± 6.1	62.5 ± 4.2	69.8 ± 5.6∗
Training experience, y	6.1 ± 2.4	5.9 ± 1.7	6.4 ± 3.0
World Athletics score, AU	978 ± 78	1000 ± 45	954 ± 96

Abbreviations: BMI, body mass index; NCAA, National Collegiate Athletic Association; VO_2_max, volume of maximal oxygen uptake.

1 The demographic questionnaire allowed participants to select multiple choices for race/ethnicity, and thus total sample sizes in males are >14.

∗ Indicates a sex difference (*P* < 0.05). Sex differences were assessed by unpaired t-tests on normally distributed data. Non-normal data were log-transformed to create a normal distribution (BMI, weight). Mann-Whitney tests were utilized when log transformation did not create a normal distribution (VO_2_max, training experience). Data are presented as untransformed means ± SDs.

### Macronutrient intake

Dietary intakes of energy, macronutrients, and adherence to the DRIs are presented in Table 2. Guidelines from ACSM/AND/DC encourage endurance athletes to consume carbohydrate intakes of 6–10 g/kg bodyweight per day (g·kg^–1^·d^–1^) [3]. Across the competitive season, 43% of females and 29% of males met this guideline, where dietary carbohydrate intake was 5.67 ± 1.16 g·kg^–1^·d^–1^ in females and 4.95 ± 1.05 g·kg^–1^·d^–1^ in males (main effect of sex, *P* = 0.0960). Protein intake was 2.09 ± 0.425 g·kg^–1^·d^–1^ in females and 1.92 ± 0.519 g·kg^–1^·d^–1^ in males (*P* = 0.357). All participants met the minimum protein intake guideline for athletes (>1.2 g·kg^–1^·d^–1^), and most females (57%) and nearly half of the males (43%) consumed ≥2.0 g·kg^–1^·d^–1^ recommended for athletes at risk for energy deficiency [3].

**TABLE 2:** Dietary intake and dietary reference intake adherence in NCAA Division I cross country student-athletes during a competitive season.

Nutrients	Combined	Females	Males	Combined	Females	Males
Energy and macronutrients	Mean ± SD	Mean ± SD	Mean ± SD	%DRI adherence	%DRI adherence	%DRI adherence
Energy intake, kcal	2450 ± 490	2270 ± 467	2620 ± 463	--	--	--
Carbohydrates, g/d	300 ± 63.8	287 ± 68.5	312 ± 57.3	100	100	100
Carbohydrates, %kcal^1^	49.1 ± 4.92	50.5 ± 5.59	47.7 ± 3.87	82	86	79
Protein, g/d	113 ± 27.9	107 ± 29.3	120 ± 25.6	100	100	100
Protein, %kcal^1^	18.6 ± 2.69	18.7 ± 2.81	18.4 ± 2.66	100	100	100
Fat, g/d	91.5 ± 22.9	82.4 ± 19.7	100 ± 22.9∗	--	--	--
Fat, %kcal^1^	33.6 ± 4.29	32.8 ± 5.06	34.4 ± 3.37	60	57	64
Linoleic acid, g/d^2^	17.7 ± 4.42	15.7 ± 2.78	19.6 ± 5.00∗	82	100	64
Linoleic acid, %kcal^1^	6.53 ± 1.10	6.32 ± 0.774	6.74 ± 1.35	96	100	92
α-Linolenic acid, g/d^2^	1.64 ± 0.537	1.64 ± 0.608	1.65 ± 0.479	71	93	50
α-Linolenic acid, %kcal^1^	0.624 ± 0.243	0.683 ± 0.312	0.565 ± 0.131	43	36	50
Cholesterol, mg/d	359 ± 142	314 ± 117	405 ± 154	--	--	--
Fiber, g/d^2^	25.3 ± 8.77	29.6 ± 9.19	21.0 ± 5.96∗	29	57	0
Vitamins	Mean ± SD	Mean ± SD	Mean ± SD	%DRI adherence	%DRI adherence	%DRI adherence
Vitamin A, μg/d^3^	941 ± 353	1130 ± 310	755 ± 297∗	64	93	36
Thiamin, mg/d	2.89 ± 1.53	3.52 ± 1.94	2.26 ± 0.510	100	100	100
Riboflavin, mg/d	2.68 ± 0.794	2.99 ± 0.800	2.38 ± 0.686∗	96	100	93
Niacin, mg/d^4^	47.6 ± 12.0	43.3 ± 12.0	51.8 ± 10.8	100	100	100
Vitamin B_6_, mg/d	3.65 ± 1.02	3.67 ± 1.14	3.64 ± 0.917	100	100	100
Folate, μg/d^5^	830 ± 326	962 ± 384	697 ± 187∗	96	93	100
Vitamin B_12_, μg/d	8.34 ± 2.41	7.79 ± 2.41	8.88 ± 2.36	100	100	100
Vitamin C, mg/d	102 ± 60.3	128 ± 68.5	77.3 ± 38.8∗	50	64	36
Vitamin D, μg/d^6^	5.03 ± 2.57	5.14 ± 1.78	4.91 ± 3.24	0	0	0
Vitamin E, mg/d^7^	13.1 ± 5.60	15.3 ± 6.30	11.0 ± 3.97∗	36	50	21
Vitamin K, μg/d^2^	174 ± 105	200 ± 105	148 ± 102	68	86	50
Choline, mg/d^2^	412 ± 122	398 ± 101	425 ± 143	29	36	21
Minerals	mean ± SD	mean ± SD	mean ± SD	%DRI adherence	%DRI adherence	%DRI adherence
Calcium, mg/d	1120 ± 313	1220 ± 307	1010 ± 296	57	79	36
Copper, *μ*g/d	1670 ± 584	1940 ± 617	1400 ± 410∗	89	93	86
Iron, mg/d	20.0 ± 5.41	20.1 ± 5.77	20.0 ± 5.23	86	71	100
Magnesium, mg/d	419 ± 127	467 ± 136	371 ± 99.8∗	60	79	43
Phosphorus, mg/d	1790 ± 433	1820 ± 457	1760 ± 422	100	100	100
Potassium, mg/d^2^	3130 ± 717	3230 ± 688	3020 ± 757	57	79	36
Selenium, *μ*g/d	144 ± 33.3	129 ± 29.2	159 ± 30.9∗	100	100	100
Sodium, mg/d^2^	4070 ± 946	3680 ± 583	4470 ± 1090∗	100	100	100
Zinc, mg/d	15.7 ± 4.34	15.9 ± 5.23	15.6 ± 3.42	96	100	93

Samples sizes are *n* = 14 for females and *n* =14 for males.

Abbreviations: AI, Adequate Intake Level; AMDR, Acceptable Macronutrient Distribution Range; DRI, Dietary Reference Intake; NCAA, National Collegiate Athletic Association; RDA, Recommended Daily Allowance.

DRI adherence was assessed by the RDA, except

1 indicates by AMDR

2 indicates by AI.

3 As retinol activity equivalents (RAEs). 1 RAE = 1 *μ*g retinol, 12 *μ*g β-carotene, 24 *μ*g α-carotene, or 24 *μ*g β-cryptoxanthin.

4 As niacin equivalents (NEs). 1 mg niacin = 60 mg tryptophan.

5 As dietary folate equivalents (DFEs). 1 DFE = 1 *μ*g food folate = 0.6 *μ*g of folic acid from fortified food or as a supplement consumed with food = 0.5 *μ*g of a supplement consume on an empty stomach.

6 As cholecalciferol. 1 *μ*g cholecalciferol = 40 IU vitamin D. The RDA for vitamin D is assessed under the assumption of minimal sunlight exposure.

7 As α-tocopherol.

∗ Indicates a sex difference (*P* < 0.05). Sex differences were assessed by unpaired t-tests on normally distributed data. Data were log-transformed to create a normal distribution (protein, %kcal; linoleic acid, g/d; vitamin E; vitamin K, folate). Mann-Whitney tests were used when log transformation did not create a normal distribution (α-linolenic acid, %kcal; thiamin). Data are presented as untransformed means ± SDs.

All participants met the RDAs for carbohydrates, protein, and AMDR for protein. Participants who did not meet the AMDR for carbohydrates (1 female, 3 males) consumed below guidelines, whereas those who did not meet the AMDR for total fat (6 females, 5 males) consumed above guidelines. All females met the AMDR and AI for linoleic acid; 92% of males met the AMDR, and 64% met the AI. For α-linolenic acid, all but 1 female and half of males met the AI, whereas only 36% of females and half of males met the AMDR. Participants who did not meet the AMDR for α-linolenic acid consumed below it, except 2 females exceeded it. For total dietary fiber, 57% of females but no males met the RDA.

### Micronutrient intake

Dietary intakes of micronutrients and adherence to the DRIs are also presented in Table 2; micronutrient intakes from supplements are provided in Supplemental Table 2. ACSM/AND/DC guidelines emphasize meeting the DRIs for vitamin D, calcium, and iron, particularly in athletes at risk for energy deficiency [3]. No participants met the RDA for vitamin D intake from diet alone. One female supplemented with ∼1000 *μ*g/d or ∼10-fold the UL. For calcium, 79% of females but only 36% of males met the RDA from diet alone. Two females and 2 males reported supplementing with calcium (∼50–100 mg/d), but supplemental calcium did not impact adherence to the DRIs. For iron, 71% of females and all males met the RDA from diet alone; however, all but 1 female and 79% of males reported supplementing with iron such that mean total iron intakes were above the UL in both females (110 ± 60.1 mg/d) and males (66.8 ± 36.3 mg/d, *P* = 0.029). Including dietary and supplemental iron, 64% of males and all but 1 female consumed above the UL (>45 mg/d).

Most or all participants met the DRIs for thiamin, riboflavin, niacin, vitamin B_6_, folate, vitamin K, copper, phosphorus, selenium, sodium, and zinc. Most females but not males met the DRIs for vitamin A, vitamin C, vitamin E, magnesium, and potassium. Less than half of the participants met the AI for choline. Dietary insufficiency of vitamin A, vitamin C, vitamin E, magnesium, potassium, and choline are consistent with a recent report in the greater United States population [32]. Supplementation with other micronutrients was reported in only 3 females and 3 males and included electrolyte mixes (2 females, 2 males), a daily multivitamin (1 female), a B-complex multivitamin (1 male), beet powder (1 female, 1 male), and a preworkout mix (1 male). One female reported supplemental folate intake >UL. No other micronutrients were consumed above the UL. All participants consumed Gatorade/Powerade nearly daily. Recovery protein beverages (e.g., Muscle Milk) were also consumed frequently. Sports and recovery drinks were not considered supplements for this study and were included in dietary intake data.

### Diet quality according to HEI-2020

**FIGURE 2:**
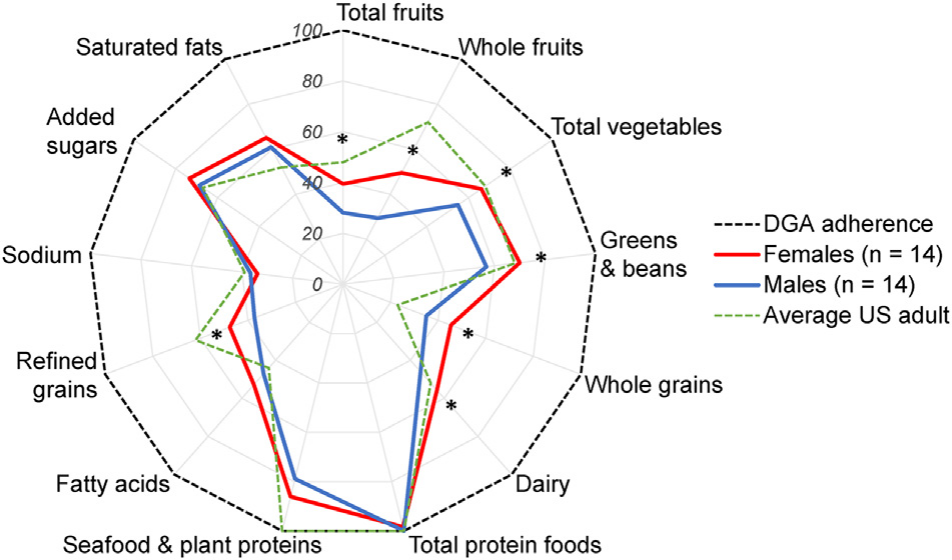
HEI-2020 scores as percent adherence to the DGAs in female and male NCAA Division I cross country student-athletes. HEI-2020 was calculated using the simple scoring algorithm. Scores are expressed as mean percent adherence to the DGAs, where a maximum score indicates 100% adherence to the DGAs. ∗Indicates a sex difference (*P* < 0.05). Sex differences were assessed by unpaired t-tests on normally distributed data. Data were log-transformed to create a normal distribution (total fruits). Mann-Whitney tests were used when log transformation did not create a normal distribution (greens and beans, whole fruit, total protein foods, seafood and plant proteins, and sodium). DGAs, Dietary Guidelines for Americans; HEI-2020, 2020 Healthy Eating Index; NCAA, National Collegiate Athletic Association.

HEI-2020 scores are plotted by percent adherence to the DGAs in Figure 2. Overall diet quality across the competitive season was poor (composite score: 57.9 ± 14.0) but consistent with the average United States adult (mean = 57) [33]. Diet quality was higher in females (65.3 ± 13.7) than in males (50.6 ± 10.1, *P* = 0.0034), as males did not score higher than females for any component. Females scored higher than males for total fruits (females: 2.56 ± 1.67, males: 1.41 ± 1.56, *P* = 0.0343), whole fruits (3.46 ± 1.72, 1.47 ± 1.65, *P* = 0.0045), total vegetables (3.89 ± 1.21, 2.75 ± 0.926, *P* = 0.0099), greens and beans (4.19 ± 1.44, 2.84 ± 1.53, *P* = 0.0237), whole grains (5.36 ± 2.48, 3.50 ± 1.64, *P* = 0.0283), dairy (6.69 ± 1.83, 4.60 ± 1.74, *P* = 0.0047), and moderation in refined grains (5.98 ± 2.75, 3.72 ± 1.72, *P* = 0.0164). Scores were similar between females and males for total protein foods (females: 4.84 ± 0.325, males: 5, *P* = 0.0840), seafood and plant proteins (4.67 ± 0.749, 3.94 ± 1.45, *P* = 0.114), fatty acids (5.85 ± 2.70, 4.71 ± 2.09, *P* = 0.224), and moderation in sodium (3.11 ± 2.82, 3.67 ± 2.70, *P* = 0.598), added sugars, (7.87 ± 1.55, 6.86 ± 2.15, *P* = 0.166), and saturated fats (6.84 ± 2.47, 6.09 ± 2.03, *P* = 0.386).

### Energy expenditure, energy balance, and training patterns

**FIGURE 3:**
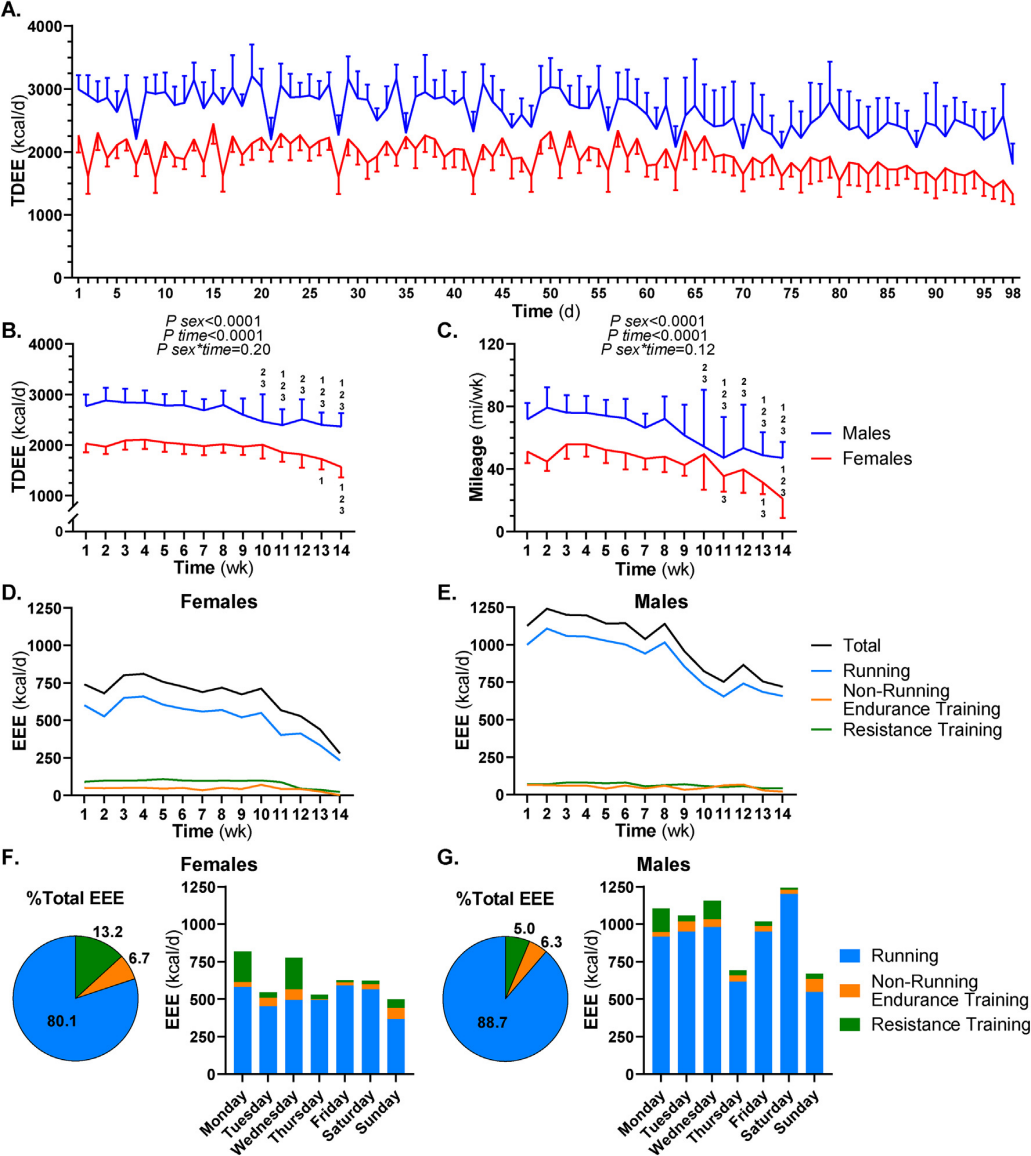
Estimated energy expenditure in NCAA Division I cross country student-athletes during a competitive season. REE was calculated using the Mifflin-St Jeor equation, and EEE was estimated for each day from training logs using the compendium of physical activities. TDEE was calculated as the sum of REE and EEE. In females and males, mean ± SD TDEE was calculated for each training day (A) and week (B), and mean ± SD running miles were calculated for each week (C). Differences in weekly TDEE and mileage were assessed by 2-way repeated measures ANOVAs with main effects of sex, time, and the sex∗time interaction. One-way repeated measures ANOVAs with Bonferroni’s post hoc comparisons assessed time differences within a sex. Numerical superscripts indicate differences compared to weeks 1, 2, and 3 (*P* < 0.05). For each training week, mean ± SD EEE was estimated for running, nonrunning endurance training, and resistance training in females (D) and males (E). Across the entire season, the percent contributions to total EEE for running, nonrunning endurance training, and resistance training and their distribution by day of the week were determined in females (F) and males (G). ANOVA, analysis of variance; EEE, exercise energy expenditure; NCAA, National Collegiate Athletic Association; REE, resting energy expenditure; TDEE, total daily energy expenditure.

Average TDEE was calculated for females and males across each training day (Figure 3A) and week (Figure 3B), and average running mileage was calculated for each training week (Figure 3C). TDEE and running mileage were greater in males than females, and both decreased during the championship season. Despite sex differences in TDEE (females: 2230 ± 139 kcal/d, males: 2840 ± 152 kcal/d) and running mileage (44.6 ± 9.86 mi/wk, 64.3 ± 11.9 mi/wk, *P* *sex* < 0.0001 for both), average daily training time did not differ between females (76.5 ± 11.1 min/d) and males (82.9 ± 18.0 min/d, *P* = 0.265). In both sexes, running contributed the most to EEE compared to other training modes (Figure 3D–G). EEE distribution by day of the week reflected training schedules, where resistance training sessions mostly occurred on Mondays and Wednesdays, and recovery days were Sundays (Figure 3F and G).

**FIGURE 4:**
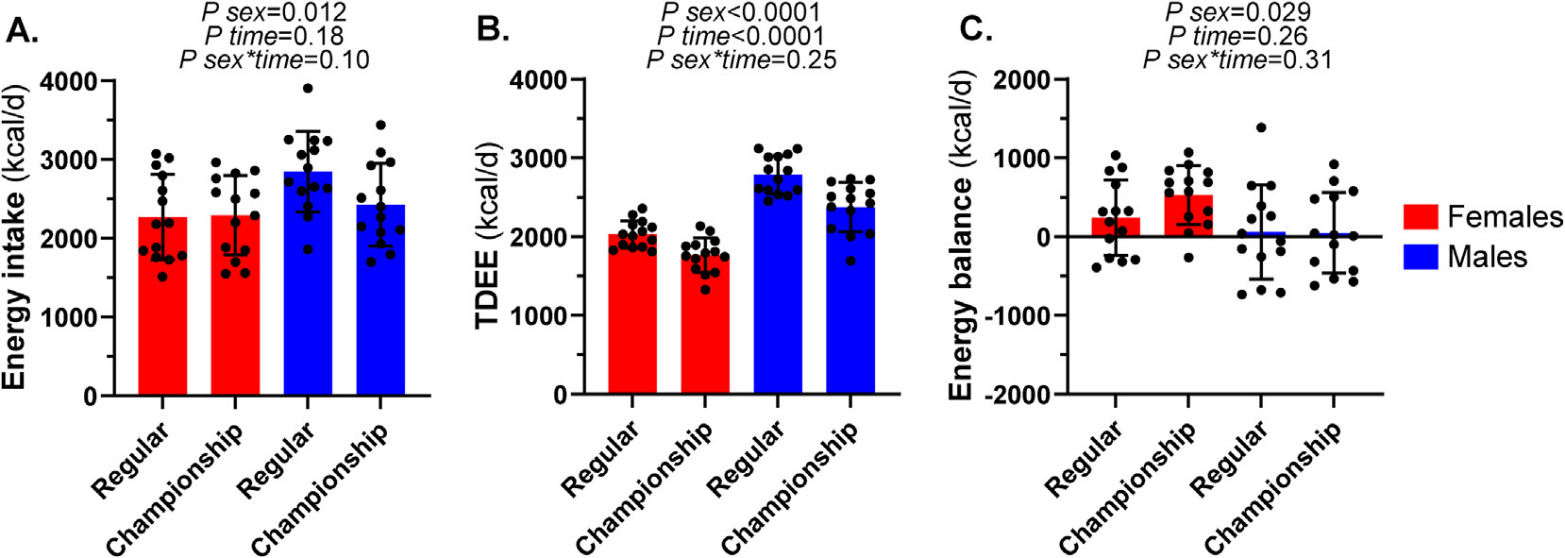
Mean energy intake, TDEE, and energy balance during the regular and championship seasons in NCAA Division I cross country student-athletes. Energy intake (A), TDEE (B), and energy balance (C) were averaged across the regular (9 wk, 4 d of diet records) and championship seasons (5 wk, 5 d of diet records). TDEE was subtracted from energy intake to calculate energy balance. Two-way repeated measures ANOVAs assessed differences in energy intake, TDEE, and energy balance by sex, time, and the sex∗time interaction. Data are means ± SDs. ANOVA, analysis of variance; NCAA, National Collegiate Athletic Association; TDEE, total daily energy expenditure.

Crude estimates of energy intake, TDEE, and balance during the regular and championship seasons are given in Figure 4. During the regular season, 36% of females and 50% of males were estimated to be in negative energy balance. During the championship season, TDEE decreased, and energy intake was maintained compared to the regular season, and only 1 female and 43% of males were estimated to be in negative energy balance. One female and 5 males reported nonseason-ending injuries or illnesses that led to minor disturbances to their training. No time or sex∗time effects were observed for bodyweight or BMI across the competitive season (*P* > 0.223 for all).

## Discussion

The current study characterized dietary intake, diet quality, and energy expenditure during a competitive season in female and male NCAA Division I cross country runners from a single school. On average, carbohydrate intakes were insufficient, but protein and fat intakes met sport-specific guidelines. Of micronutrients emphasized for athletes, no participants met the RDA for vitamin D from diet alone; most females but not males met the RDA for calcium; and a high degree of iron supplementation was observed such that total iron intakes were above the UL. HEI-2020 scores indicated poor quality diets according to the DGAs, and diet quality was greater in females than males. Crude estimates of energy balance indicated that approximately one-third of females and half of males were energy deficient during the regular season. With a decrease in training volume, energy deficiency prevalence decreased in the championship season to 46% of males and no females.

### Carbohydrate intake

ACSM/AND/DC guidelines encourage endurance athletes training 1–3 h/d to consume 6–10 g·kg^–1^·d^–1^ of carbohydrate [3]. This guideline was appropriate for the current group given that average daily training time was >1 h/d in all but 1 female (58.6 min/d) and 2 males (59.1 and 52.7 min/d). Only 43% of females and 29% of males met this guideline for carbohydrate intake, with average intakes below 6 g·kg^–1^·d^–1^ in both groups. Endogenous carbohydrate stores are finite (75 kg male, liver glycogen: ∼400–480 kcal, muscle glycogen: ∼1200–2800 kcal) [34], and muscle glycogen depletion during exercise is one of the leading factors associated with fatigue [35]. Consequently, some runners may have experienced performance impairments in training and competition due to inadequate fueling with carbohydrates. Despite generally not meeting recommendations for athletes, all participants met the RDA for absolute carbohydrate intake (130 g/d), and the few consuming outside the AMDR were above it, highlighting the greater fueling requirements for endurance athletes compared to the general population.

A limited number of studies report carbohydrate intakes, specifically in NCAA Division I cross country runners. Tanaka et al. [16] (1995) reported carbohydrate intakes of 7.9 ± 2.2 and 6.1 ± 1.3 g·kg^–1^·d^–1^ in 14 male and 10 female Division I runners [16], and Niekamp and Baer [17] (1995) reported ∼7.5 ± 2.2 g·kg^–1^·d^–1^ in 12 male Division I runners. These earlier studies suggest adequate carbohydrate intakes in Division I runners, yet more recent studies assessing diets of runners [9] and student-athletes from a range of different sports [7,8,11] indicate most Division I student-athletes do not consume sufficient carbohydrates, even when evaluated against the lower recommendation of 5–7 g·kg^–1^·d^–1^ for athletes in more moderate training programs (∼1 h/d) [3]. These studies and ours support implementing strategies to reduce the prevalence of insufficient carbohydrate intake in NCAA Division I student-athletes. One consideration is to provide education and improve food availability to promote carbohydrate intake in the hours preceding and following training sessions: 2 fueling strategies with evidence to support athletic performance [3,[36], [37], [38]]. Whole food carbohydrates high in fiber may also be healthful options given that total fiber intakes were below the RDA (females: 25–26 g/d, males: 38 g/d). The race distance for Division I cross country runners does not necessitate carbohydrate intake during the competition (females: 5 and 6 km, males: 8 and 10 km); however, carbohydrate intake during training may also be a useful fueling strategy for sessions >90 min or when multiple sessions are performed per day, though some beneficial adaptations may occur from training with low carbohydrate availability [3,[36], [37], [38]].

### Protein intake

To support the increased metabolic demand, muscle repair and remodeling, and protein turnover associated with training and competition, most athletes and active adults are recommended to consume elevated protein intakes (1.2–2.0 g·kg^–1^·d^–1^) [3] compared to the United States population (RDA = 0.8 g·kg^–1^·d^–1^) [1]. In contrast to our initial hypothesis, average protein intakes were near the higher end of this range, with no females and only 5 males consuming <1.5 g·kg^–1^·d^–1^ (minimum = 1.3 g·kg^–1^·d^–1^). We initially hypothesized insufficient protein intakes as a consequence of insufficient total energy intake; however, studies in Division I cross country runners [9,16,17] and other athletes [7,8,10,11] also support adequate protein intakes in most Division I student-athletes. In the current study, 8 females and 6 males consumed >2.0 g·kg^–1^·d^–1^ (maximums: females 2.7 g·kg^–1^·d^–1^, males 2.9 g·kg^–1^·d^–1^). Protein intakes >2.0 g·kg^–1^·d^–1^ may be encouraged for athletes, particularly if dietary carbohydrate or energy deficiencies are likely during an intensified training period [3]. It is possible that higher protein intakes in this subset of runners were protective or even compensatory for periods of insufficient carbohydrate or energy intake. For runners with energy deficiency, increasing both carbohydrate and protein intake is recommended to prevent energy deficiency and its consequences on short-term athletic performance and long-term health [[39], [40], [41]]. In the current study, runners with positive energy balance may consider replacing some protein with carbohydrates to ensure athlete guidelines are met for both macronutrients. Protein quality and intake timing should also be considered. Strategies such as consuming larger protein boluses with adequate leucine content following exercise and prior to sleep are thought to be advantageous for muscle recovery and adaptation to training [3,38].

### Fat intake

ACSM/AND/DC recommendations are to consume fat intakes within age-specific AMDRs (ages 14–18 y: 25–35% kcal, >19 y: 20–35% kcal) [3]. The rationale for this recommendation is that current evidence does not support performance benefits related to high-fat diets, especially when fat is consumed at the expense of carbohydrates [37,42], and restricting fat intake increases the risk for deficiencies in other nutrients, such as fat-soluble vitamins [3]. Similar to prior reports in both Division I cross country runners [9,16,17] and student-athletes from other sports [7,8,11], average fat intakes met or exceeded the AMDR. Considering the need to increase carbohydrate intake and given that fat intakes were toward the high end or exceeded the AMDR, foods high in fat may be worth substituting for healthy sources of dietary carbohydrates. Consuming better-quality fats may be important, as the current study and others indicate inadequate intakes of omega (ω)-3 polyunsaturated fats and poor moderation of saturated fats in Division I student-athletes [7,[9], [10], [11], [12],^18^]. For athletes with energy deficiency, increasing carbohydrate and protein intakes without decreasing absolute fat intakes may also improve total fat AMDR adherence.

### Micronutrient intake

To support bone health during strenuous training, ACSM/AND/DC guidelines indicate that athletes should meet or exceed the RDAs for vitamin D (15 *μ*g/d) and calcium (ages 14–18 y: 1300 mg/d, >19 y: 1000 mg/d) [3], a recommendation important for cross country runners due to a higher prevalence of stress fractures compared to other collegiate student-athletes, particularly in females [43]. Mean vitamin D intakes were approximately one-third of the RDA, with no individuals meeting the RDA and only 1 consuming >10 *μ*g/d from diet alone. For calcium, over three-quarters of females but only half of males met the RDA from diet alone. Vitamin D and calcium intakes below the RDA are also reported in other Division I student-athletes [[8], [9], [10],^17^]. Randomized-controlled trials in United States military recruits show some efficacy in vitamin D and calcium supplementation to prevent bone injury during strenuous training [[44], [45], [46]]. In the current study, calcium supplementation was negligible, and 1 female consumed a vitamin D supplement to help meet the RDA. Vitamin D intakes below the RDA may not necessarily indicate compromised status in the current study. The RDA is set with an assumption of minimal sunlight exposure [1]. The runners in the current study trained predominantly outdoors at ∼30.4°N latitude, below where athletes are considered at increased risk for deficiency [3]. Other factors, including sunlight exposure, sunscreen use, and skin pigmentation, should also be considered when evaluating vitamin D intake requirements [47].

Declines in iron status are frequently reported in endurance athletes and other physically active populations and can impact performance, mostly attributable to iron’s rate-limiting effect on hemoglobin synthesis and oxygen-carrying capacity in the blood [48]. In agreement with recommendations for endurance athletes [3], mean dietary intakes exceeded the RDA in both females (15–18 mg/d) and males (8–11 mg/d). Moreover, all but 1 female and 3 males reported supplementing with iron such that mean intakes exceeded the UL (45 mg/d) in both sexes when including supplements. We previously characterized this group as nonanemic with low iron stores, according to hemoglobin and serum ferritin collected at baseline [19]. A possible reason for low iron stores despite intakes >UL is inhibition of dietary iron absorption by the iron-regulatory hormone hepcidin [19,48]. Improving carbohydrate and energy intakes may improve iron absorption in runners, as elevations in hepcidin are attributed to muscle glycogen depletion during exercise and chronic energy deficiency [[48], [49], [50]]. Consuming more bioavailable heme-iron and vitamin C with nonheme iron are also 2 strategies to improve iron absorption [51].

### Diet quality

According to HEI-2020 scores, the diets of runners in the current study poorly adhered to the DGAs, with greater diet quality in females than males. Other studies utilizing the current or prior HEIs also indicate poor quality dietary patterns in Division I student-athletes [[10], [11], [12]]. Poor diets in college students are not surprising. For most students, college coincides with the transition from adolescence to young adulthood, a period associated with declining intakes of fruits, vegetables, dairy, and whole grains and higher intakes of fast food and sugar-sweetened beverages [[52], [53], [54], [55]]. In the current study and others [12], possible reasons for poorer diet quality in males may be that male student-athletes are more likely to consume fast food and dine out, whereas females are more likely to prepare their meals [56]. Other barriers to healthful diets in collegiate student-athletes include poor access to grocery stores, home kitchens, and nutrient-dense foods on campus [10,57]. HEI-2020 component scores were especially low for total and whole fruits, total vegetables, greens and beans, whole grains, dairy, and fatty acids (*see above*). Fruits, vegetables, and whole grains are foods dense in carbohydrates, fiber, and many vitamins and minerals. Plant foods high in protein (beans, legumes, and nuts) are also typically high-quality sources of fat, containing ω-3 polyunsaturated fats. Dairy is a rich source of calcium and is considered a high-quality source of protein [58]. Increasing intakes of these foods in Division I cross country runners may both improve intakes of nutrients important to athletes and encourage a dietary pattern in line with the DGAs.

HEI-2020 component scores also indicate that the runners in the current study were nearly adherent to the DGAs for total protein foods and seafood and plant proteins, whereas moderation scores were poor for refined grains, added sugars, sodium, and saturated fats. Recommendations based on some of these findings may not necessarily support health and performance in Division I cross country runners, given their unique nutritional requirements. For instance, component scores for total protein foods and seafood and plant proteins are evaluated based on consuming sufficient protein foods to meet the RDA for protein [2] but do not consider the higher protein recommendations for athletes [3]. Thus, maximum scores for these components do not ensure athletes are meeting their higher protein intake guidelines. Moreover, refined grain and added sugar intakes greater than what is recommended for the general population may help runners meet their elevated carbohydrate and energy needs. Similarly, higher intakes of sodium and other electrolytes may help replace sweat losses during training [59], especially given the warm training climate of the runners in the current study. Such considerations indicate that the HEI-2020 is not specifically designed for the dietary intake requirements of competitive athletes [24], and validated dietary assessment tools specific to athletic populations are lacking [60].

In conclusion, the current study contributes to the literature as one of the more comprehensive analyses of dietary intakes in collegiate student-athletes and one of very few reporting diets of female and male Division I cross country student-athletes (reviewed in Supplemental Table 1). A strength is the evaluation of nutrients in foods using USDA databases [22,23], which enabled intake estimates of a wider number of nutrients than other studies in collegiate student-athletes. Moreover, using FPED allowed an estimate of diet quality by the HEI-2020, which no study has reported exclusively in Division I cross country runners. Another strength is the collection of ∼9 d of food records estimating dietary intake across the entire competitive season, whereas other studies in Division I student-athletes utilize fewer food records, single timepoints, or rely on surveys of habitual intake rather than assessing intakes of specific foods as part of a food record. However, it is also important to note that the current study was not primarily designed to monitor diet, where food record collection was a secondary aim, with timing chosen around primary outcomes. Food records also have inherent limitations [28], although few other methods are capable of assessing *ad libitum* dietary intakes in applied settings. Considering this study’s other limitations, future studies should also assess the longitudinal impacts of diet on competitive performance and biomarkers of nutritional status and health. We assessed correlations between diet, VO_2_max, and nonphysiological performance variables (e.g., average race finish on the team, season-best race time, Supplemental Materials) but found no meaningful relationships, likely due to a small sample size. Additionally, future studies should characterize intakes of other nutrients not reported in FNDDS or SR-Legacy, such as individual amino acids, carotenoids, and fatty acids, while also providing more precise and objective estimates of energy expenditure using doubly-labeled water. We are hopeful that the recent changes to NCAA legislation have a positive impact on the diets of Division I student-athletes, but the legislation does not address student-athletes from Divisions II, III, junior colleges, and club sports, who have less funding and fewer nutrition resources [56]. Nevertheless, we hope the current study informs administrators, coaches, and sports dietitians on possible dietary needs in Division I cross country runners and serves as a template for larger dietary analyses of collegiate student-athletes.

## Author contributions

The authors’ responsibilities were as follows– DEB, CEB, SRH: conceived and designed the study; DEB: led data collection, analysis, and manuscript preparation with oversight from SRH; DEB, ARH: analyzed food records; SNC, CEB: calculated HEI-2020 scores; and all authors: read and approved the final manuscript. All authors were affiliated with Florida State University during data collection. All other affiliations are current to the manuscript submission date.

## Funding

This work was supported by intramural funds to SRH from Florida State University and Pennington Biomedical Research Center and a grant to DEB and SRH from the Atlantic Coast Conference Innovation Initiative.

## Data availability

Data described in the manuscript are made freely available in the following Mendeley dataset https://doi.org/10.17632/r8z6bbcmn3.1. Data are also available by request to DEB. The analytical code for the Healthy Eating Index-2020 scores is made publicly available by the National Cancer Institute at https://epi.grants.cancer.gov/hei/sas-code.html.

## Conflict of interest

The authors report no conflicts of interest. Views expressed do not necessarily reflect the positions and policies of the United States Army Research Institute of Environmental Medicine, United States Army, or Department of Defense.
