# Supplementary material for: Dietary Intake and Diet Quality of Female and Male NCAA Division I Cross Country Runners from a Single University
Source: Curr Dev Nutr. 2024 Oct 15;8(11):104475. doi: 10.1016/j.cdnut.2024.104475 (PMC11602982; doi:10.1016/j.cdnut.2024.104475)
Supplement: multimedia component 1 [file mmc1.docx]

**Supplemental Table 1.** Studies of dietary intake in NCAA Division I student-athletes.

| **Ref.**  **(#)** | **Population** | **Diet assessment methods** | **ACSM/AND/DC guidelines** | **DRIs, DGAs** | **Additional notes** |
| --- | --- | --- | --- | --- | --- |
| **Shriver et al.**  **2013 (7)** | Females  *n* = 45  45% soccer  40% basketball  16% cross country or track & field | 3-d multi-pass diet recalls during pre-season/early competition | **Energy**: 1939 ± 604 kcal/d  91% in energy deficit  **CHO**: 4.0 ± 1.0 g∙kg^-1^∙d^-1^  **PRO**: 1.2 ± 0.4 g∙kg^-1^∙d^-1^ | **CHO** (g/d, %kcal):  257 ± 77, 54 ± 6  **PRO** (g/d, %kcal):  77 ± 22, 16 ± 3  **Fat** (g/d, %kcal):  69 ± 29, 31 ± 6 | Reports eating habits assessed by questionnaire |
| **Danh et al.**  **2023 (8)** | Females  *n* = 14  Volleyball players | 3-d food records administered at pre-season, during-season, and post-season (12 d total) | **Energy**: 1791 ± 450 kcal/d  Group average energy deficit  **CHO***: 3.0 ± 0.9 g∙kg^-1^∙d^-1^  **PRO***: 1.3 ± 0.5 g∙kg^-1^∙d^-1^  **Ca^**: 764 (366, 959) g/d  **Vit D**: 3.43 ± 2.28 μg/d  **Fe^**: 14 (10, 18) mg/d | **CHO**: 217 ± 64 g/d  **PRO**: 92 ± 35 g/d  **Fat**: 64 ± 19 g/d  **SFA**: 22 ± 7 g/d  **Cholesterol**: 358 ± 196 g/d | 6 participants also provided 24-h food record during off season, which are analyzed separately in the paper  Reports intakes of vitamins A, B_6_, B_12_, C, D, thiamin, riboflavin, niacin, folate, phosphorus, potassium, sodium  Assesses sports nutrition knowledge with questionnaire |
| **Beerman et al.**  **2020 (9)** | Females & males  *n* = 21 (M), 20 (F)  Cross country runners | Food frequency questionnaire | **Energy** (kcal/d):  M: 2742 ± 815, F: 1927 ± 638  **CHO** (g∙kg^-1^∙d^-1^):  M: 5.3 ± 1.7, F: 4.7 ± 1.9  **PRO*** (g∙kg^-1^∙d^-1^):  M: 1.9 ± 0.1, F: 1.2 ± 0.1  **Ca** (mg/d):  M: 1358 ± 352, F: 965 ± 399  **Vit D** (μg/d):  M: 6.9 ± 0.7, F: 3.1 ± 2.2  **Fe** (mg/d):  M: 23.3 ± 6.3, F: 15.2 4.9 | **CHO** (%kcal):  M: 50.7 ± 5.9, F: 51.0 ± 6.2  **PRO** (%kcal):  M: 17.1 ± 2.0, F: 15.2 ± 1.8  **Fat** (%kcal):  M: 33.7 ± 5.0, F: 35.2 ± 4.7  **SFA** (%kcal):  M: 10.9 ± 2.3, F: 11.1 ± 2.1 | Calculates energy availability based on lean body mass  Reports intakes of vitamins A, B_12_, C, potassium, sodium, and zinc  Also reports micronutrient intakes with and without supplements |
| **Gieng et al.**  **2023 (10)** | Females  *n* = 41  Soccer, *n* = 13  Swimming, *n* = 12  Basketball, *n* = 4  Cross country, *n* = 2  Gymnastics, *n =* 10 | 3-d multi-pass diet recalls during competitive season | **PRO**: 1.4 ± 0.5 g∙kg^-1^∙d^-1^  **Ca** (mg/d):  ages 14-18 y: 1076 ± 200  ages 19-30 y: 958 ± 398  **Vit D**: 4.4 ± 2.6 μg/d | **LA**: 18.3 ± 6.7 g/d  **ALA**: 1.9 ± 0.8 g/d  **HEI-2015**: 56.2 ± 13.5 | Assesses dietary inflammatory index and bone mineral density |
| **Werner et al.**  **2022 (11)** | Females & males  *n* = 94 (22% female)  Cross country runners (14% of males and 21% of females) | 1-d multi-pass diet recall, administered between March-June 2020 when students were off-campus and not participating in team activities | **Energy** (kcal/d):  M: 3299 ± 1513, F: 2224 ± 727  **CHO*** (g∙kg^-1^∙d^-1^):  M: 4.2 ± 2.0, F: 3.8 ± 1.5  **PRO*** (g∙kg^-1^∙d^-1^):  M: 2.1 ± 1.0, F: 1.4 ± 0.5 | **CHO** (g/d):  M: 336 ± 161, F: 264 ± 105  **PRO** (g/d):  M: 170 ± 82, F: 97 ± 36  **Fat** (g/d):  M: 145 ± 70, F: 91 ± 38  **HEI-2015**: 59.2 ± 16.6 | No significant differences in HEI-2015 between sexes, class, majors, sports played, or between those who did or did not report taking previous nutrition coursework  *Bodyweight not reported. CHO and PRO intake relative to BW were conservatively estimated using 70 and 80 kg for females and males, respectively. |
| **Jontony et al.**  **2020 (12)** | Females & males  *n* = 129  F rowing, *n* = 56  F swimming, *n* = 17  F gymnastics, *n* = 7  M swimming, *n* = 19  M wrestling, *n* = 7 |  |  | **HEI-2015**:  Group mean: 71.0 ± 11.2  F rowing: 73.5 ± 9.7  F swimming: 72.8 ± 10.8  F gymnastics: 68.9 ± 12.9  M swimming: 68.2 ± 12.7  M wrestling: 56.5 ± 5.7 | Reports skin carotenoid status |
| **Tanaka et al.**  **1995 (16)** | Females & males  *n* = 14 (M), 10 (F)  Cross country runners | 4-d food records administered once during the training season and once during competition (8 d total) | **Energy** (kcal/d):  M: 3586 ± 208, F: 1967 ± 150  **CHO** (g∙kg^-1^∙d^-1^):  M: 8.0 ± 0.6, F: 6.1 ± 0.4  **PRO*** (g∙kg^-1^∙d^-1^):  M: 1.9 ± 0.1, F: 1.2 ± 0.1 | **CHO** (g/d, %kcal):  M: 506 ± 36, 56 ± 2  F: 329 ± 21, 68 ± 3  **PRO** (g/d, %kcal):  M: 124 ± 8, 14 ± 1  F: 64 ± 5, 13 ± 1  **Fat** (g/d, %kcal):  M: 113 ± 7, 29 ± 1  F: 49 ± 7, 22 ± 2  **Fiber** (g/d):  M: 18 ± 2, F: 17 ± 2 | Give differences in dietary intake between a training and competition period |
| **Niekamp et al.**  **1995 (17)** | Males  *n* = 12  Cross country runners | 4-d food records administered once during the training season and once during competition (8 d total) | **Energy**: 3248 ± 589 kcal/d  **CHO***: 7.5 ± 2.0 g∙kg^-1^∙d^-1^  **PRO***: 1.6 ± 0.4 g∙kg^-1^∙d^-1^  **Ca**: 1340 ± 414 mg/d  **Fe**: 18.1 ± 4.9 mg/d | **CHO**: 497 ± 134 g, 61% kcal  **PRO**: 104 ± 24 g, 13% kcal  **Fat**: 92 ± 29 g, 26% kcal | Reports intakes of vitamins A, B_6_, C, thiamin, riboflavin, niacin, folate, magnesium, and zinc |
| **Ritz et al.**  **2020 (18)** | Females & males  *n* = 1528 (49% female)  19.8% football  80.2% other (includes cross country) | Food frequency questionnaire |  | **ALA**: 62 (4%) met the RDA | Blood analysis for omega-3 status |

Studies were included that reported intake of one or more nutrients in the DRIs and/or HEI scores. Data are means ± SDs, except ^ indicates median (IQR). *Indicates that intake relative to bodyweight was estimated by dividing average absolute intakes by average bodyweights. ACSM, American College of Sport Medicine; ALA, α-linolenic acid; AND, Academy of Nutrition and Dietetics; BW, bodyweight; Ca, calcium; CHO, carbohydrate; DC, Dietitians of Canada; DRIs, Dietary Reference Intakes; Fe, iron; HEI, Healthy Eating Index; LA, linoleic acid; NCAA, National Collegiate Athletic Association; PRO, protein; SFA, saturated fat.

**Supplemental Table 2.** Micronutrient intake from supplements and prevalence of use in NCAA Division I cross country student-athletes across a competitive season.

| **Micronutrients** | **All participants** (14 females, 14 males) | | | **Supplement users only** | | |
| --- | --- | --- | --- | --- | --- | --- |
|  | **Combined** | **Females** | **Males** | **Combined** | **Females** | **Males** |
| **Vitamins**: | *mean ± SD* | *mean ± SD* | *mean ± SD* | *n*, *mean ± SD* | *n*, *mean ± SD* | *n*, *mean ± SD* |
| **Vitamin A**, µg/d ^a^ | 69.4 ± 367 | 139 ± 520 | 0 | 1, 1940 | 1, 1940 | 0, 0 |
| **Vitamin C**, mg/d | 8.27 ± 23.9 | 4.27 ± 11.2 | 12.3 ± 32.1 | 4, 57.9 ± 40.7 | 2, 29.9 ± 9.31 | 2, 86.9 ± 21.2 |
| **Vitamin D**, µg/d ^b^ | 34.1 ± 181 | 68.3 ± 255 | 0 | 1, 956 | 1, 956 | 0, 0 |
| **Vitamin E**, mg/d ^c^ | 0.279 ± 1.48 | 0.558 ± 2.09 | 0 | 1, 7.82 | 1, 7.82 | 0, 0 |
| **Vitamin K**, µg/d ^#^ | 0 | 0 | 0 | 0, 0 | 0, 0 | 0, 0 |
| **Thiamin**, mg/d | 0 | 0 | 0 | 0, 0 | 0, 0 | 0, 0 |
| **Riboflavin**, mg/d | 0.0810 ± 0.428 | 0 | 0.162 ± 0.606 | 1, 2.27 | 0, 0 | 1, 2.27 |
| **Niacin**, mg/d ^d^ | 1.46 ± 4.44 | 0.814 ± 3.05 | 2.11 ± 5.55 | 3, 13.6 ± 4.13 | 1, 11.4 | 2, 14.8 ± 5.15 |
| **Vitamin B_6_**, mg/d | 0.154 ± 0.410 | 0.193 ± 0.496 | 0.116 ± 0.317 | 4, 1.08 ± 0.611 | 2, 1.35 ± 0.294 | 2, 0.809 ± 0.427 |
| **Folate**, µg/d ^e^ | 11.1 ± 58.8 | 22.2 ± 83.1 | 0 | 1, 311 | 1, 311 | 0, 0 |
| **Vitamin B_12_**, µg/d | 119 ± 630 | 0.744 ± 2.02 | 238 ± 891 | 4, 836 ± 1490 | 2, 5.21 ± 2.53 | 2, 1670 ± 2360 |
| **Choline**, mg/d ^#^ | 0 | 0 | 0 | 0, 0 | 0, 0 | 0, 0 |
| **Minerals**: | *mean ± SD* | *mean ± SD* | *mean ± SD* | *n*, *mean ± SD* | *n*, *mean ± SD* | *n*, *mean ± SD* |
| **Calcium**, mg/d | 6.56 ± 21.9 | 8.10 ± 28.7 | 5.01 ± 12.7 | 4, 45.9 ± 43.5 | 2, 56.7 ± 72.2 | 2, 35.1 ± 2.44 |
| **Copper**, µg/d | 0 | 0 | 0 | 0, 0 | 0, 0 | 0, 0 |
| **Iron**, mg/d | 68.4 ± 51.5 | 90.0 ± 57.5 | 46.8 ± 34.7 | 24, 79.8 ± 46.6 | 13, 96.9 ± 53.4 | 11, 59.5 ± 27.0 |
| **Magnesium**, mg/d ^f^ | 1.00 ± 3.41 | 0.405 ± 1.51 | 1.60 ± 4.59 | 3, 9.33 ± 6.35 | 1, 5.67 | 11.2 ± 7.78 |
| **Phosphorus**, mg/d | 1.53 ± 8.08 | 3.06 ± 11.43 | 0 | 1, 42.8 | 1, 42.8 | 0, 0 |
| **Potassium**, mg/d ^#^ | 10.5 ± 38.1 | 13.6 ± 49.3 | 7.47 ± 23.6 | 4, 73.8 ± 82.6 | 2, 95.3 ± 127 | 52.3 ± 50.4 |
| **Selenium**, µg/d | 0 | 0 | 0 | 0, 0 | 0, 0 | 0, 0 |
| **Zinc**, mg/d | 0.0694 ± 0.367 | 0.139 ± 0.520 | 0 | 1, 1.94 | 1, 1.94 | 0, 0 |

^a^As retinol activity equivalents (RAEs). 1 RAE = 1 μg retinol, 12 μg β-carotene, 24 μg α-carotene, or 24 μg β-cryptoxanthin. ^b^As cholecalciferol. 1 µg cholecalciferol = 40 IU vitamin D. ^c^As α-tocopherol. ^d^As niacin equivalents (NEs). 1 mg niacin = 60 mg tryptophan. ^e^As dietary folate equivalents (DFEs). 1 DFE = 1 μg food folate = 0.6 μg of folic acid from fortified food or as a supplement consumed with food = 0.5 μg of a supplement taken on an empty stomach.

**Dietary Recall**

**Friday 9/27/19**

| Food Item  (give ingredients) | Brand | Amount | Cooking Method  Did you use oil?  Did you use seasoning? | Time Consumed |
| --- | --- | --- | --- | --- |
|  |  |  |  |  |
|  |  |  |  |  |
|  |  |  |  |  |
|  |  |  |  |  |
|  |  |  |  |  |
|  |  |  |  |  |
|  |  |  |  |  |
|  |  |  |  |  |
|  |  |  |  |  |
|  |  |  |  |  |
|  |  |  |  |  |
|  |  |  |  |  |
|  |  |  |  |  |
|  |  |  |  |  |
|  |  |  |  |  |
|  |  |  |  |  |
|  |  |  |  |  |
|  |  |  |  |  |
|  |  |  |  |  |
|  |  |  |  |  |
|  |  |  |  |  |

**Saturday 9/28/19**

| Food Item  (give ingredients) | Brand | Amount | Cooking Method  Did you use oil?  Did you use seasoning? | Time Consumed |
| --- | --- | --- | --- | --- |
|  |  |  |  |  |
|  |  |  |  |  |
|  |  |  |  |  |
|  |  |  |  |  |
|  |  |  |  |  |
|  |  |  |  |  |
|  |  |  |  |  |
|  |  |  |  |  |
|  |  |  |  |  |
|  |  |  |  |  |
|  |  |  |  |  |
|  |  |  |  |  |
|  |  |  |  |  |
|  |  |  |  |  |
|  |  |  |  |  |
|  |  |  |  |  |
|  |  |  |  |  |
|  |  |  |  |  |
|  |  |  |  |  |
|  |  |  |  |  |
|  |  |  |  |  |
|  |  |  |  |  |

**Sunday 9/29/19**

| Food Item  (give ingredients) | Brand | Amount | Cooking Method  Did you use oil?  Did you use seasoning? | Time Consumed |
| --- | --- | --- | --- | --- |
|  |  |  |  |  |
|  |  |  |  |  |
|  |  |  |  |  |
|  |  |  |  |  |
|  |  |  |  |  |
|  |  |  |  |  |
|  |  |  |  |  |
|  |  |  |  |  |
|  |  |  |  |  |
|  |  |  |  |  |
|  |  |  |  |  |
|  |  |  |  |  |
|  |  |  |  |  |
|  |  |  |  |  |
|  |  |  |  |  |
|  |  |  |  |  |
|  |  |  |  |  |
|  |  |  |  |  |
|  |  |  |  |  |
|  |  |  |  |  |
|  |  |  |  |  |
|  |  |  |  |  |

**
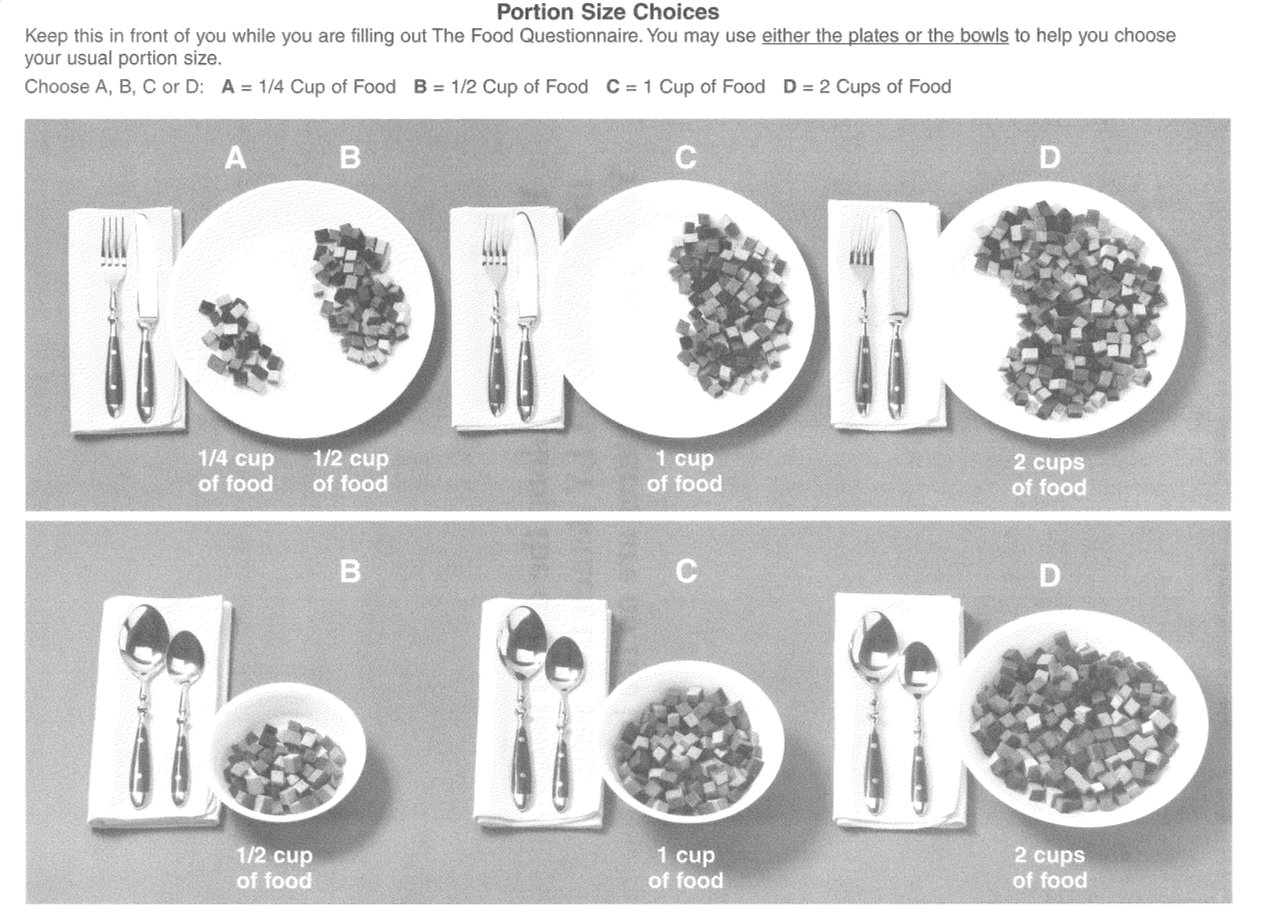
**

**Qualtrics^TM^ Training Habit Questionnaire**

1. What was your average weekly mileage during the following types of training weeks? If you did not train during a time period, please enter the number “0”.

Regular season, non-race week: _____

Regular season, race week: _____

Conferences, race week: _____

Non-race week after conferences: _____

Regionals race week: _____

Nationals race week: _____

1. How many miles were your easy or regular runs (NOT your low mileage recovery or shakeout runs) during the following types of training weeks? If you did not train during a time period, enter the number “0”.

Regular season, non-race week: _____

Regular season, race week: _____

Conferences, race week: _____

Non-race week after conferences: _____

Regionals race week: _____

Nationals race week: _____

1. What was your running pace on a typical easy or regular run (NOT your low mileage recovery or shakeout runs)? Please select one.

10 min/mile

9 min/mile

8.5 min/mile

8 min/mile

7.5 min/mile

7 min/mile

6.5 min/mile

6 min/mile

5.5 min/mile

Other, please specify if selected: _____

1. What was the distance of a typical long run during the following types of training weeks? If you did not train during a time period, please enter the number “0”.

Regular season, non-race week: _____

Regular season, race week: _____

Conferences, race week: _____

Non-race week after conferences: _____

Regionals race week: _____

Nationals race week: _____

1. On a typical long run, what pace would you run? Please select one.

10 min/mile

9 min/mile

8.5 min/mile

8 min/mile

7.5 min/mile

7 min/mile

6.5 min/mile

6 min/mile

5.5 min/mile

Other, please specify if selected: _____

1. On a typical workout day, how many miles would you warm-up prior to the workout? Please select one.

1 mile

1.5 miles

2 miles

2.5 miles

3 miles

3.5 miles

Other, please specify if selected: _____

1. On a typical workout day, what pace would you run for the warm-up? Please select one.

10 min/mile

9 min/mile

8.5 min/mile

8 min/mile

7.5 min/mile

7 min/mile

6.5 min/mile

6 min/mile

5.5 min/mile

Other, please specify if selected: _____

1. On a typical workout day, how many miles would you cool-down prior to the workout? Please select one.

1 mile

1.5 miles

2 miles

2.5 miles

3 miles

3.5 miles

Other, please specify if selected: _____

1. On a typical workout day, what pace would you run for the cool-down? Please select one.

10 min/mile

9 min/mile

8.5 min/mile

8 min/mile

7.5 min/mile

7 min/mile

6.5 min/mile

6 min/mile

5.5 min/mile

Other, please specify if selected: _____

1. During the following types of training weeks, how many double runs would you perform in each week? For example, if you typically ran double runs on 2 days per week during the regular season, enter the number “2” in the appropriate space.

Regular season, non-race week: _____

Regular season, race week: _____

Conferences, race week: _____

Non-race week after conferences: _____

Regionals race week: _____

Nationals race week: _____

1. On a typical double run, how many miles would you run? Please select one.

1 mile

1.5 miles

2 miles

2.5 miles

3 miles

3.5 miles

4 miles

4.5 miles

5 miles

5.5 miles

6 miles

Other, please specify if selected: _____

1. On a typical double run, what pace would you run? Please select one.

10 min/mile

9 min/mile

8.5 min/mile

8 min/mile

7.5 min/mile

7 min/mile

6.5 min/mile

6 min/mile

5.5 min/mile

Other, please specify if selected: _____

1. During the following types of training weeks, how many low mileage recovery runs would you perform in each week? For example, if you typically ran a low mileage recovery run on 2 days per week during the regular season, enter the number “2” in the appropriate space.

Regular season, non-race week: _____

Regular season, race week: _____

Conferences, race week: _____

Non-race week after conferences: _____

Regionals race week: _____

Nationals race week: _____

1. On a typical low mileage recovery run, how many miles would you run? Please select one.

1 mile

1.5 miles

2 miles

2.5 miles

3 miles

3.5 miles

4 miles

4.5 miles

5 miles

6.5 miles

6 miles

Other, please specify if selected: _____

1. On a typical low mileage recovery run, what pace would you run? Please select one.

10 min/mile

9 min/mile

8.5 min/mile

8 min/mile

7.5 min/mile

7 min/mile

6.5 min/mile

6 min/mile

5.5 min/mile

Other, please specify if selected: _____

1. During the following types of training weeks, how many miles would you run for a typical premeet run?

Regular season, non-race week: _____

Regular season, race week: _____

Conferences, race week: _____

Non-race week after conferences: _____

Regionals race week: _____

Nationals race week: _____

1. On a typical premeet run, what pace would you run? Please select one.

10 min/mile

9 min/mile

8.5 min/mile

8 min/mile

7.5 min/mile

7 min/mile

6.5 min/mile

6 min/mile

5.5 min/mile

Other, please specify if selected: _____

1. During the following types of training weeks, how many miles would you warm-up for a race?

Regular season, non-race week: _____

Regular season, race week: _____

Conferences, race week: _____

Non-race week after conferences: _____

Regionals race week: _____

Nationals race week: _____

1. On a typical race warm-up, what pace would you run? Please select one.

10 min/mile

9 min/mile

8.5 min/mile

8 min/mile

7.5 min/mile

7 min/mile

6.5 min/mile

6 min/mile

5.5 min/mile

Other, please specify if selected: _____

1. During the following types of training weeks, how many miles would you cool-down following a race?

Regular season, non-race week: _____

Regular season, race week: _____

Conferences, race week: _____

Non-race week after conferences: _____

Regionals race week: _____

Nationals race week: _____

1. On a typical race cool-down, what pace would you run? Please select one.

10 min/mile

9 min/mile

8.5 min/mile

8 min/mile

7.5 min/mile

7 min/mile

6.5 min/mile

6 min/mile

5.5 min/mile

Other, please specify if selected: _____

1. During the following types of training weeks, how many days off would you take (no running or cross training) if you weren’t injured?

Regular season, non-race week: _____

Regular season, race week: _____

Conferences, race week: _____

Non-race week after conferences: _____

Regionals race week: _____

Nationals race week: _____

1. During the following types of training weeks, how many times per week would you perform each of the listed modes of cross training?

Regular season, non-race week

Road bike: _____

[*shown if selected*] How much time in minutes was a typical session? _____

Stationary bike: _____

[*shown if selected*] How much time in minutes was a typical session? _____

Swim: _____

[*shown if selected*] How much time in minutes was a typical session? _____

Elliptical: _____

[*shown if selected*] How much time in minutes was a typical session? _____

Rowing machine: _____

[*shown if selected*] How much time in minutes was a typical session? _____

Aqua jog / pool running: _____

[*shown if selected*] How much time in minutes was a typical session? _____

Other, please specify activity and frequency: _____

[*shown if selected*] How much time in minutes was a typical session? _____

Regular season, race week

Road bike: _____

[*shown if selected*] How much time in minutes was a typical session? _____

Stationary bike: _____

[*shown if selected*] How much time in minutes was a typical session? _____

Swim: _____

[*shown if selected*] How much time in minutes was a typical session? _____

Elliptical: _____

[*shown if selected*] How much time in minutes was a typical session? _____

Rowing machine: _____

[*shown if selected*] How much time in minutes was a typical session? _____

Aqua jog / pool running: _____

[*shown if selected*] How much time in minutes was a typical session? _____

Other, please specify activity and frequency: _____

[*shown if selected*] How much time in minutes was a typical session? _____

Regular season, race week

Road bike: _____

[*shown if selected*] How much time in minutes was a typical session? _____

Stationary bike: _____

[*shown if selected*] How much time in minutes was a typical session? _____

Swim: _____

[*shown if selected*] How much time in minutes was a typical session? _____

Elliptical: _____

[*shown if selected*] How much time in minutes was a typical session? _____

Rowing machine: _____

[*shown if selected*] How much time in minutes was a typical session? _____

Aqua jog / pool running: _____

[*shown if selected*] How much time in minutes was a typical session? _____

Other, please specify activity and frequency: _____

[*shown if selected*] How much time in minutes was a typical session? _____

Conferences, race week

Road bike: _____

[*shown if selected*] How much time in minutes was a typical session? _____

Stationary bike: _____

[*shown if selected*] How much time in minutes was a typical session? _____

Swim: _____

[*shown if selected*] How much time in minutes was a typical session? _____

Elliptical: _____

[*shown if selected*] How much time in minutes was a typical session? _____

Rowing machine: _____

[*shown if selected*] How much time in minutes was a typical session? _____

Aqua jog / pool running: _____

[*shown if selected*] How much time in minutes was a typical session? _____

Other, please specify activity and frequency: _____

[*shown if selected*] How much time in minutes was a typical session? _____

Non-race week after conferences

Road bike: _____

[*shown if selected*] How much time in minutes was a typical session? _____

Stationary bike: _____

[*shown if selected*] How much time in minutes was a typical session? _____

Swim: _____

[*shown if selected*] How much time in minutes was a typical session? _____

Elliptical: _____

[*shown if selected*] How much time in minutes was a typical session? _____

Rowing machine: _____

[*shown if selected*] How much time in minutes was a typical session? _____

Aqua jog / pool running: _____

[*shown if selected*] How much time in minutes was a typical session? _____

Other, please specify activity and frequency: _____

[*shown if selected*] How much time in minutes was a typical session? _____

Regionals race week

Road bike: _____

[*shown if selected*] How much time in minutes was a typical session? _____

Stationary bike: _____

[*shown if selected*] How much time in minutes was a typical session? _____

Swim: _____

[*shown if selected*] How much time in minutes was a typical session? _____

Elliptical: _____

[*shown if selected*] How much time in minutes was a typical session? _____

Rowing machine: _____

[*shown if selected*] How much time in minutes was a typical session? _____

Aqua jog / pool running: _____

[*shown if selected*] How much time in minutes was a typical session? _____

Other, please specify activity and frequency: _____

[*shown if selected*] How much time in minutes was a typical session? _____

Nationals race week

Road bike: _____

[*shown if selected*] How much time in minutes was a typical session? _____

Stationary bike: _____

[*shown if selected*] How much time in minutes was a typical session? _____

Swim: _____

[*shown if selected*] How much time in minutes was a typical session? _____

Elliptical: _____

[*shown if selected*] How much time in minutes was a typical session? _____

Rowing machine: _____

[*shown if selected*] How much time in minutes was a typical session? _____

Aqua jog / pool running: _____

[*shown if selected*] How much time in minutes was a typical session? _____

Other, please specify activity and frequency: _____

[*shown if selected*] How much time in minutes was a typical session? _____

1. [*shown if road bike was selected in Question 23*] At what speed was a typical road bike session? Please refer to effort levels if you do not know your speed.

10-11.9 mph, slow light effort

12-13.9 mph, moderate effort

14-15.9 mph, fast vigorous effort

16-19 mph, very vigorous

1. [*shown if stationary bike was selected in Question 23*] At what wattage was a typical stationary bike session? Please refer to effort levels if you do not know your wattage.

30-50 watts, very light to light effort

51-89 watts, light-to-moderate effort

90-100 watts, moderate effort

101-160 watts, vigorous effort

161-200 watts, very vigorous effort

201-270 watts, extremely vigorous effort

1. [*shown if swim was selected in Question 23*] How would you best describe the intensity of your typical swim session?

Slow, light, or moderate effort

Fast or vigorous effort

1. [*shown if rowing machine was selected in Question 23*] At what wattage was a typical rowing session? Please refer to effort levels if you do not know your wattage.

<100 watts, light effort

100 watts, moderate effort

150 watts, vigorous effort

≥200 watts, very vigorous effort

1. During the following types of training weeks, how many days per week would you perform core exercises? If you did not perform core during a time period, please enter the number “0”.

Regular season, non-race week: _____

Regular season, race week: _____

Conferences, race week: _____

Non-race week after conferences: _____

Regionals race week: _____

Nationals race week: _____

1. How would you best describe the intensity at which you did core?

Light

Moderate

Vigorous

I did not perform core during the 2019 cross country season.

1. During the following types of training weeks, how many days per week would you lift weights? If you did not lift weights during a time period, please enter the number “0”.

Regular season, non-race week: _____

Regular season, race week: _____

Conferences, race week: _____

Non-race week after conferences: _____

Regionals race week: _____

Nationals race week: _____

1. How would you best describe a typical weightlifting session?

Low weight, high repetitions (8-15 repetitions)

Heavy weights (e.g., squatting)

I did not lift weights during the 2019 cross country season.
